# Comparative effects of modified rotary scarf osteotomy and traditional scarf osteotomy in treating moderate to severe hallux valgus: a retrospective cohort study

**DOI:** 10.1186/s12891-023-07156-5

**Published:** 2024-01-13

**Authors:** Zi Li, Weiwei Yu, Shiwei Lin, Ke Fu, Zhenhua Fang

**Affiliations:** Department of Orthopaedics, Wuhan Fourth Hospital, No. 473 Hanzheng Street, Qiaokou District, Wuhan, Hubei China

**Keywords:** Comparative study, Hallux valgus, Osteotomy, Scarf osteotomy

## Abstract

**Background:**

Traditional Scarf osteotomy (TSO) is an effective procedure with a good record in moderate to severe hallux valgus (MSHV) surgery. In order to overcome shortcomings of TSO, Modified Rotary Scarf osteotomy (MRSO) was introduced in this study, which aimed to compare the clinical and radiological outcome in the patients treated with MRSO or TSO.

**Methods:**

Of 175 patients (247 feet) with MSHV, 100 patients (138 feet) treated with MRSO and 75 patients (109 feet) treated with TSO were evaluated according to relevant indicators in twenty-four months follow-up. Pre-surgical and post-surgical HVA, IMA, DMAA, MTP-1 ROM, sesamoid grade and AOFAS (American Orthopaedic Foot and Ankle Society) scores and postsurgical complications were evaluated.

**Results:**

Both groups manifested similar baseline characters. The mean follow-up was of 25.9 (range, 22–37) months. Significantly lower IMA, lower Sesamoid grade and higher DMAA at six months, twelve months and twenty-four months post-surgically had been showed in MRSO group compared to TSO group. There was no significant difference in HVA, MTP-1 ROM and AOFAS data at each follow-up time point post-surgically between the two groups. No major complications occurred in either group.

**Conclusion:**

MRSO showed comparable results to TSO, and improved IMA and sesamoid grade to a greater extent, with a lower probability of throughing effect. Although DMAA could be increased by MRSO, MRSO could still be a reproducible, non-dangerous and efficacious alternative procedure for treating HV patients which do not have severe DMAA.

## Introduction

Compared with surgical treatment, conservative treatment (foot orthoses and minimalist running interventions) can better restore the biomechanical gait function o in hallux valgus (HV) patients [1], but for cases with obvious symptoms or even transfer metatarsalgia, non-surgical or exercise intervention may not be appropriate [2–5]. Medium to serious hallux valgus abnormity is considered as hallux valgus angle (HVA) over 30 degrees or 1st-2nd-intermetatarsal angle (IMA) over 13 degrees [6]. Currently, there are many bone surgeries to treat HV, including open surgeries such as Chevron osteotomy, Scarf osteotomy or Lapidus surgery and minimally invasive surgeries such as Reverdin-Isham osteotomy [7], Intramedullary Nail Device surgery [8]. The above surgical techniques have all achieved satisfactory results, but which technique has the best results has yet to be determined [9]. Scarf osteotomy is the most commonly performed diaphyseal osteotomy for the correction of medium or serious HV and gained a good reputation [10–13].

In TSO, the IMA is reduced by shifting the plantar metatarsal osteotomy bone segment laterally to increase the load on the first metatarsal row [14, 15], and the dorsal metatarsal osteotomy bone segment is shifted medially to correct metatarsal varus [16]. This procedure lengthens or shortens the first metatarsal, and its osteotomy stability allows the patient to bear weight early and return to activity [17, 18]. However, TSO has its own disadvantages [19–21]. The correction capability of this procedure is mainly limited by the width of the metatarsal diaphysis, i.e. the wider the diameter of metatarsal diaphysis, the greater the moving distance and the greater the correction on IMA. Because TSO moves the bone fragments transversely instead of rotating them, the postoperative IMA, DMAA (distal metatarsal articular angle) changes were limited [22]. In addition, the contact between the cortical bone and the cancellous bone after the displacement of the bone fragment makes the cortical bone insert into the cancellous bone in later postoperative term, thus the “troughing effect” [19, 23, 24], which is a common complication by TSO.

**Fig. 1:**
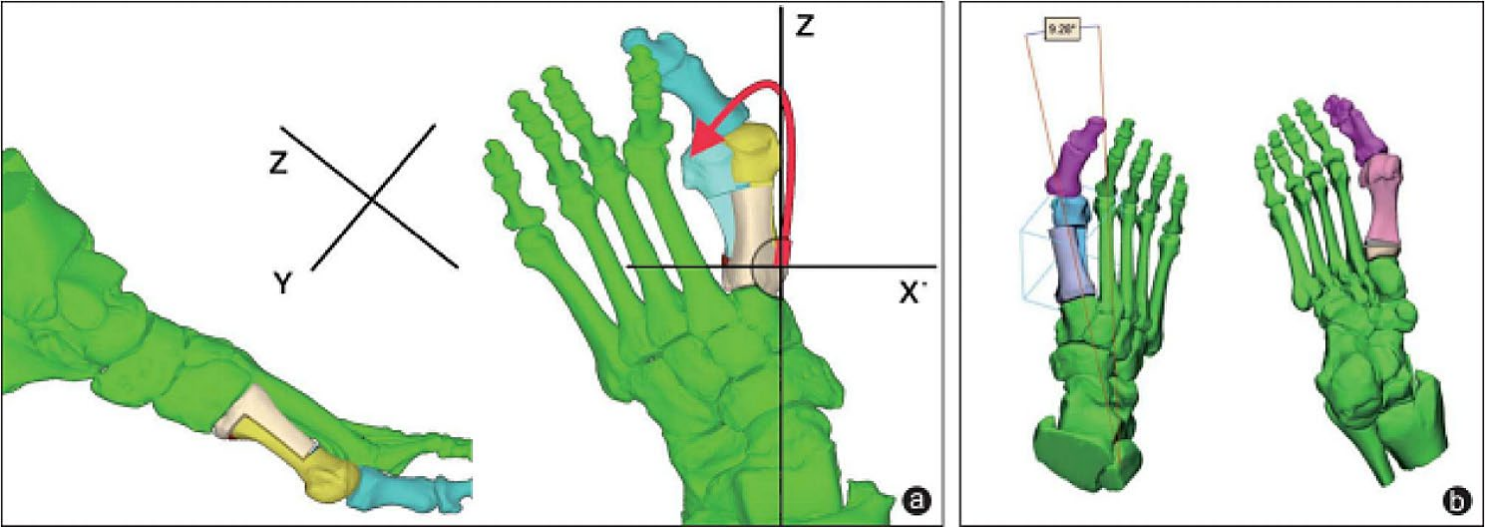
Preoperative planning. (**a**) Determine the center of rotation based on the preoperative 3D CT, and establish the X/Y/Z three-axis for preoperative planning. (**b**) The IMA angle that can be achieved after the simulated osteotomy rotation

Compared with the TSO, the MRSO retains the Z-shape of the osteotomy and applies a rotational movement (Fig. 1) instead of lateral movement of the osteotomy shaft, which has been reported to reduce the occurrence of the troughing effect and improve the IMA to a greater extent [19]. Meanwhile, the relevant conclusions need to be further demonstrated. Therefore, the objective of this backward-looking clinic trial was to investigate whether MRSO would improve the outcomes compared with TSO.

## Materials and methods

This clinic trial was permitted by the Ethics Panel of our hospital. All patients included in this randomized controlled trial signed the declaration of agreement. From March 2017 to March 2021, 175 patients (247 feet) with MSHV were underwent surgery and were retrospectively evaluated. All procedures were executed by the same senior orthopedic specialist experienced in foot surgery.

### Patient eligibility

Eligible patients were diagnosed as MSHV (HVA > 30° or IMA > 13°) and all of them initially received conservative treatment for over six months. Surgical intervention should only be considered if the patient has failed to respond to conservative treatment for no less than six months. Patients with neurovascular defects, active local infection, previous history of foot and ankle surgery, musculoskeletal inflammatory diseases (gout, rheumatism, etc.) were excluded from the study. Any concomitant surgery on first tarsal metatarsal joint or the lesser metatarsal was excluded. However, procedures performed to the lesser phalanges were not excluding factors. Patients receiving MRSO procedure were assigned to MRSO group or patients receiving TSO procedure were assigned to TSO group, and both were tracked for twenty-four months.

### Modified rotary scarf osteotomy operative technique

#### Positioning and application of tourniquet

The patient was set in dorsal recumbent position under spinal anesthesia, the drape was sterilized, and a tourniquet was applied on the upper 1/3 of the thigh after the blood was expelled.

#### Incision and osteophyte removal

A long longitudinal incision was made on the medial side of the MTT-1 (first metatarsal). Subcutaneous tissue was cut in to expose the joint capsule, then an “L”-shaped incision was made to expose the MTP-1 (first metatarsophalangeal) joint. The sagittal saw was used to remove the medial osteophyte of the MTT-1 head.

#### “Z”-shaped osteotomy and two proximal osteotomy line

**Fig. 2:**
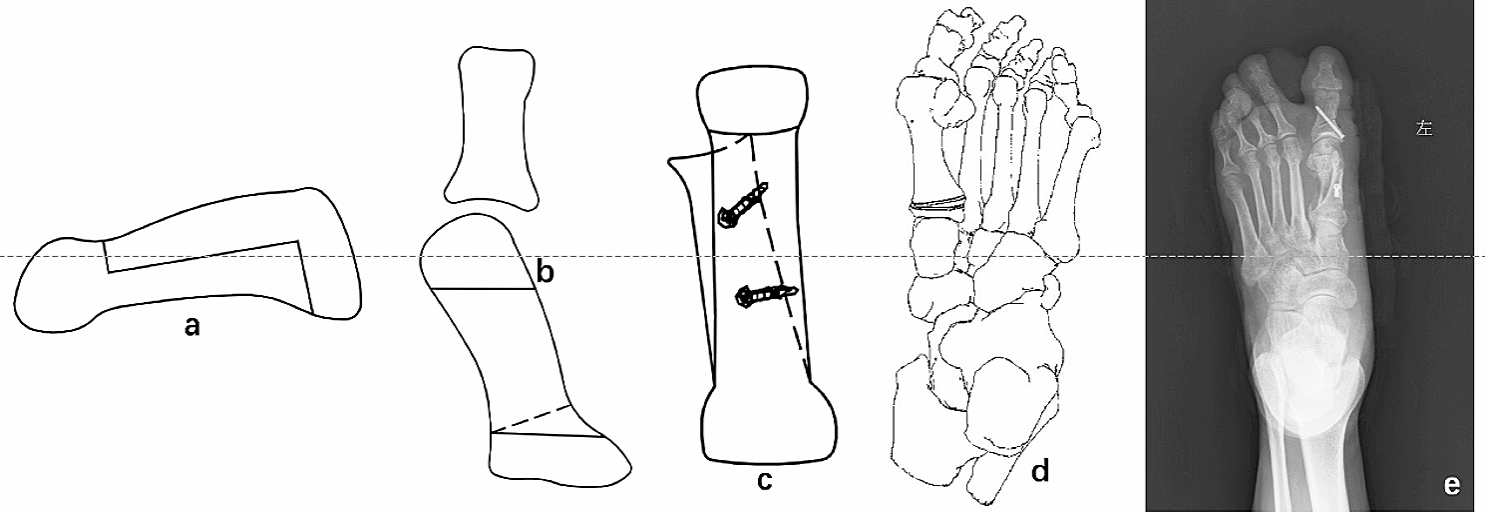
Schematic diagram of modified rotary Scarf osteotomy. (**a**) Schematic diagram of medial osteotomy of modified rotary Scarf osteotomy. (**b**) Different from traditional Scarf osteotomy, a second oblique osteotomy line (dotted line in the figure) is added to form proximal wedge-shaped bone piece. (**c**) The wedge-shaped bone piece was taken out and then rotated and moved, and the hollow screw was placed for fixation. (**d**) Schematic diagram of the wedge-shaped bone piece. (**e**) X-ray film after modified rotary Scarf osteotomy and single screw fixation

A transverse osteotomy line (first proximal osteotomy line) was marked on the plantar side of the MTT-1 bone 5 mm distal to the tarsometatarsal joint. A “Z”-shaped osteotomy was performed with a micro pendulum saw as in traditional Scarf osteotomy (Fig. 2a), and then the second proximal osteotomy line (an additional oblique osteotomy line) was made from the outer boundary to the inner boundary of the MTT-1 bone (Fig. 2b), forming a “wedge-shaped bone piece”, which was removed for later use (Fig. 2d).

#### Rotation and filling

After the osteotomy is completed, the lateral joint capsule was loosened with a sharp knife through the metatarsal shafts. The center of rotation of angulation (CORA) (i.e., the point where the two proximal osteotomy lines intersect) [25] was used as the fulcrum to rotate plantar shaft to bring the first and second proximal osteotomy lines together. When the metatarsal osteotomy shafts were rotated, a small wedge-shaped gap area appeared on the dorsal medial side of the distal metatarsal, which was filled by the spare wedge-shaped bone piece described above.

#### Fixion

After the rotation and filling was completed, 1–2 guide pins were inserted to maintain the reduction (Fig. 2e). Next, 1 or 2 hollow compression screws were fixed through the guide pins which was satisfactory under fluoroscopy (Fig. 2c).

#### Removing excess bone and filling again

The medial excess bone was removed by micro-oscillating saw to make the medial edge tidy, and above-mentioned “wedge-shaped bone piece” was filled into the groove formed by the distal osteotomy surface after rotation.

#### Additional Akin osteotomy

Finally, Akin osteotomy was performed in the first proximal phalange bone to improve the first ray alignment [10, 26].

### Postoperative treatment

After the suturing is completed, a loosely wrapped bandage is used to isolate between the 1st and 2nd toes to achieve a slight varus of the toes. Cotton pads and elastic bandages are used for dressing without external fixation with plaster or braces. On the 2nd day after the operation, patients can wear the forefoot decompression shoes and walk with weight on the ground. The walking time is determined according to the wound condition, and the stitches are removed 2 weeks after the operation. From 2 days to 4 or 6 weeks after surgery, the forefoot decompression shoes were worn to walk with weight on the ground to meet the basic needs of daily life. X-ray films were re-examined 1 month after the operation to observe the healing of the osteotomy to decide when to wear normal shoes for activities and systematic rehabilitation training.

### Observation index and curative effect evaluation

After the operation, the patients were followed up in the outpatient clinic, the clinical curative effect was evaluated, and the occurrence of complications was recorded. Basic data including age, gender, Body Mass Index (BMI) and Visual Analogue Scale (VAS) were gathered. Before operation, after operation, six, twelve, and twenty-four months post-surgically anteroposterior and lateral X-ray films of the affected foot were taken, and single person used the same method to accurately measure the position of HVA, IMA, DMAA, MTP-1 ROM (first metatarsophalangeal joint range of motion), AOFAS score and tibial sesamoid (the location of the tibial sesamoid is divided into 7 grades from the tibial border of the metatarsal head neck to the fibular border, 1–4 is normal, and 5–7 is abnormal) [27].

### Statistical analysis

Via GraphPad Prism7.0, all statistical analyzes were carried out by an independent statistician. Continuous variables are presented as mean ± standard deviation (SD), and categorical variables (tibial sesamoid, occurrence of complications) are presented numerically. Pre-surgical and post-surgical measurements were compared using a paired-sample t-test. P < 0.05 was regarded as significant difference.

## Result

**Fig. 3:**
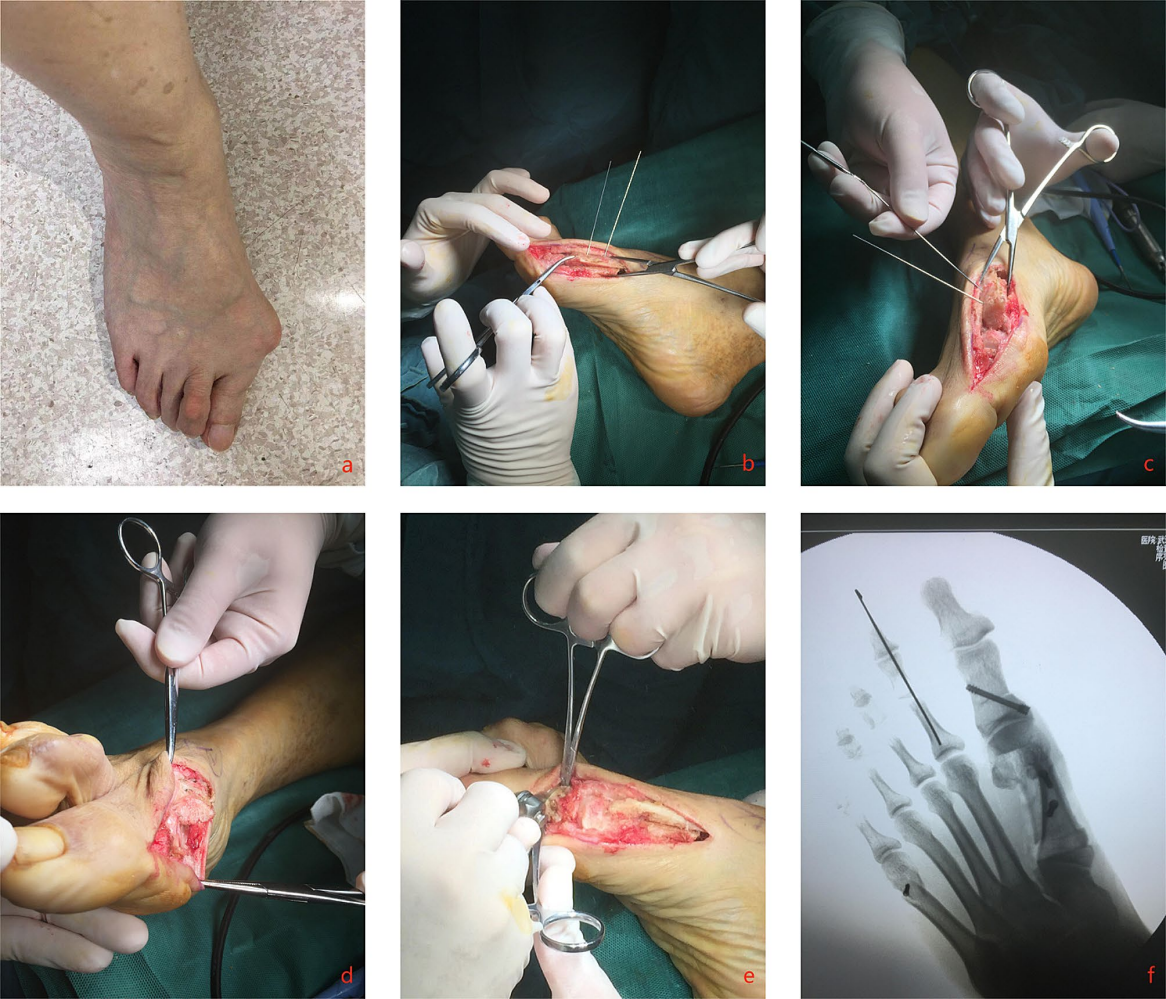
A 47-year-old female patient with severe valgus of her right foot. She underwent surgery on March 5, 2019. Postoperative follow-up showed good squatting activities and no special discomfort. (**a**) Preoperative appearance. (**b**, **c**) Distal lateral rotation after proximal wedge osteotomy. (**d**, **e**) Removal of redundant osteophytes with pendulum saw after cannulated screw fixation (**f**) postoperative fluoroscopy shows obvious changes in HVA, IMA, and DMAA

In this study, 100 patients treated with MRSO (138 feet) and 75 patients treated with TSO (109 feet) participated. Both cohorts shared the same baseline characteristics, including mean age (56.66 ± 1.3 and 48.24 ± 1.3 years, p = 0.1386), sex distribution (male: female, n, 13:87 and 11:64, p = 0.7511), BMI (kg/m^2^, 27.2 ± 4.1 and 28.7 ± 4.9, p = 0.4174) and mean VAS pain scores (88.1 and 91.6, p = 0.3959) (Table 1). Patients in the MRSO group had significantly lower IMA and higher DMAA at six months (both p < 0.0001), twelve months (both p < 0.0001) and twenty-four months (both p < 0.0001) after surgery compared to TSO group. Patients in the MRSO group had significantly lower Sesamoid grade at six months (p = 0.0171), twelve months (p = 0.0397) and twenty-four months (p = 0.0334) after surgery compared to TSO group. HVA, MTP-1 ROM and AOFAS data at each follow-up time point post-surgically in MRSO group had no significant difference compared to TSO group. Osteotomy healing within 8 weeks was observed in both groups. 4 cases of troughing and 3 cases of hallus varus were observed in TSO group, however delayed healing and non-union complications were not found in both groups (Table 2). A typical case is shown in Fig. 3.

**Table 1:** Patient’s baseline characters

	MRSO (n = 100)	TSO (n = 75)	P value
Age, y, mean + SD	56.66 ± 1.3	48.24 ± 1.3	0.1386
Gender, male: female, n	13:87	11:64	0.7511
BMI, kg/m^2^, mean + SD	27.2 ± 4.1	28.7 ± 4.9	0.4174
VAS pain score (0–100), mean	88.1	91.6	0.3959

Chi-square test was used to test gender

**Table 2:** Clinical and radiographic results

	MRSO (138 feet)	TSO (109 feet)	P value	Test
HVA before surgery	34.62 ± 0.68	33.65 ± 0.75	0.3420	Unpaired t test
HVA 6 months	3.17 ± 0.15	3.10 ± 0.14	0.5327	Unpaired t test
HVA 12 months	3.96 ± 0.11	4.23 ± 0.13	0.2234	Unpaired t test
HVA 24 months	4.18 ± 0.17	4.79 ± 0.14	0.1657	Unpaired t test
IMA before surgery	16.86 ± 1.59	17.01 ± 1.72	0.6882	Unpaired t test
IMA 6 months*	5.44 ± 0.08	8.29 ± 0.10	< 0.0001	Unpaired t test
IMA 12 months*	5.02 ± 0.06	8.78 ± 0.08	< 0.0001	Unpaired t test
IMA 24 months*	5.40 ± 0.07	9.06 ± 0.08	< 0.0001	Unpaired t test
DMAA before surgery	20.08 ± 2.21	19.69 ± 2.66	0.6684	Unpaired t test
DMAA 6 months*	30.79 ± 2.44	17.33 ± 2.87	< 0.0001	Unpaired t test
DMAA 12 months*	22.57 ± 2.19	16.78 ± 2.84	< 0.0001	Unpaired t test
DMAA 24 months*	17.68 ± 2.47	13.07 ± 1.93	< 0.0001	Unpaired t test
MTP-1 ROM (dorsiflexion) before surgery	68.68 ± 3.21	68.99 ± 3.44	0.4549	Unpaired t test
MTP-1 ROM (dorsiflexion) 6 months	69.32 ± 2.99	69.13 ± 2.74	0.4135	Unpaired t test
MTP-1 ROM (dorsiflexion) 12 months	71.56 ± 2.91	70.67 ± 2.67	0.3953	Unpaired t test
MTP-1 ROM (dorsiflexion) 24 months	73.43 ± 2.78	72.99 ± 3.03	0.3545	Unpaired t test
MTP-1 ROM (plantarflexion) before surgery	33.45 ± 3.03	33.95 ± 3.34	0.4657	Unpaired t test
MTP-1 ROM (plantarflexion) 6 months	35.51 ± 2.93	34.87 ± 3.25	0.3824	Unpaired t test
MTP-1 ROM (plantarflexion) 12 months	36.84 ± 2.98	36.03 ± 2.66	0.3392	Unpaired t test
MTP-1 ROM (plantarflexion) 24 months	37.65 ± 2.25	37.13 ± 3.04	0.2596	Unpaired t test
AOFAS before surgery	44.56 ± 3.54	44.01 ± 3.73	0.3264	Unpaired t test
AOFAS 6 months	77.43 ± 0.62	76.80 ± 0.83	0.1329	Unpaired t test
AOFAS 12 months	80.42 ± 1.79	81.04 ± 2.74	0.4023	Unpaired t test
AOFAS 24 months	80.34 ± 0.48	79.46 ± 0.84	0.1493	Unpaired t test
Sesamoid grade before surgery	2.43 ± 0.10	2.48 ± 0.09	0.3425	Unpaired t test
Sesamoid grade 6 months*	1.57 ± 0.05	2.08 ± 0.05	0.0171	Unpaired t test
Sesamoid grade 12 months*	1.45 ± 0.05	1.99 ± 0.05	0.0397	Unpaired t test
Sesamoid grade 24 months*	1.47 ± 0.06	2.01 ± 0.05	0.0334	Unpaired t test
Complication hallus varus	0	3	0.0886	Fisher’s exact test
Complication delayed healing	0	0	–	–
Complication nonunion	0	0	–	–
Complication troughing*	0	4	0.0399	Fisher’s exact test

100 patients treated with MRSO (138 feet) and 75 patients treated with TSO (109 feet)

## Discussion

The principal contribution of this clinic trial is that both MRSO and TSO surgery showed the similarly good clinical outcome in terms of HVA, AOFAS score and MTP-1 ROM. Whereas, as seen on the aspect of IMA and sesamoid grade, the MRSO procedure produced remarkably superior radiographic outcomes.

Weil and Borrelli named the Z-shaped MTT-1 osteotomy as the Scarf osteotomy [9, 28] when they studied local vascularization, modified osteotomy, and increased osteotomy length [29]. In the end, Barouk promoted the technology worldwide, especially in Europe [9]. Traditional Scarf osteotomy reduces the IMA and restores the load of the first metatarsal alignment by shifting the plantar osteotomy bone fragment laterally and shifting the dorsal metatarsal osteotomy bone fragment medially [30, 31]. Internal fixation occupies the width of the metatarsal shaft, which makes the movement of bone shafts limited, resulting in limited ability to correct deformities, so that it is suitable for mild to moderate deformities instead of severe deformity. Furthermore, its common postoperative complications are troughing effect [32], in addition to transfer metatarsalgia, undercorrection or recurrence, overcorrection, varus, degenerative arthritis, unstable fixation, and delayed union [33, 34].

**Fig. 4:**
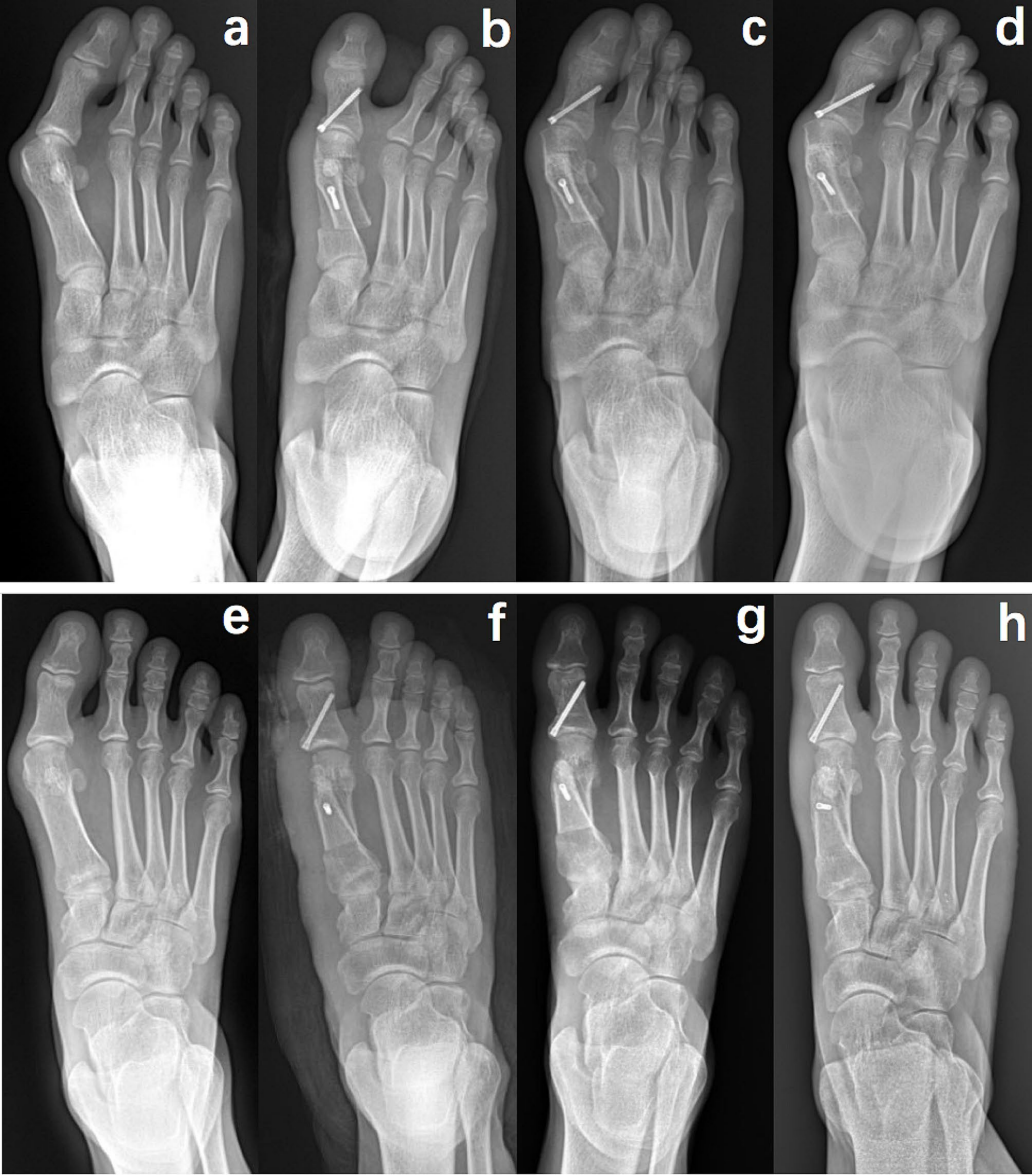
Radiological comparison between TSO and MRSO. (**a**–**d**) One person who received TSO, no significant changes in IMA and DMAA before and after operation. However, the HVA was increased one month after TSO, it relapsed four months after TSO. “**a**” represents pre-surgery; “**b**” represents post-surgery; “**d**” represents one month post-surgery; “**d**” represents six months post-surgery. (**e**–**h**) Another person who received MRSO, IMA and DMAA were obvious changed before and after surgery. “**e**” represents pre-surgery; “**f**” represents post-surgery; “**g**” represents one month post-surgery; “**h**” represents six months post-surgery. The osteotomy end heals faster in the person who received TSO compared to another person in TSO. Because of the “lock” structure that formed by the rotation in MRSO, single screw fixation does not affect its stability. Compared with double-screw fixation, the potential correction ability of single-screw is stronger, and its postoperative IMA and HVA are significantly changed

The modified rotary Scarf osteotomy uses CORA as the fulcrum to rotate the shaft outward, moving the IMA along the X-axis, raising or sinking the first metatarsal head along the Y-axis, and lengthening or shortening the length of the metatarsal along the Z-axis [35]. Meanwhile, the CORA of the rotational Scarf osteotomy is closer to the proximal end compared to the translational Scarf osteotomy, resulting in a better ability to correct IMA, DMAA [36] (Fig. 4). Because of the existence of the wedge-shaped osteotomy, no matter whether the length of the metatarsal is shortened or lengthened, the proximal end of the metatarsal tends to be complete (keep alignment between bone fragments), and the total overlapping area of the osteotomy surface is larger, which makes the biomechanics more stable and the healing speed faster [37]. We choose to make two osteotomy lines on the plantar side of the proximal MTT-1 to produce a wedge-shaped bone piece, instead of producing that on the dorsal side of the distal MTT-1, which could result in necrosis or subsidence of the metatarsal head and arthritis of the first metatarsophalangeal [38].

Establishing X/Y/Z three-axis surgical planning based on foot weight-bearing X-ray films and three-dimensional CT before operation can make the position and degree of MRSO more accurate. According to the establishment of the preoperative Y/Z axis and forefoot metatarsalgia condition, the position of the metatarsal head can be restored during the operation by adjusting the osteotomy direction such as plantarward or dorsarward, inward or outward [17], and the condition of the flat foot or high arch can also be improved [16, 39]. Because of the proximal wedge osteotomy and the determination of CORA, this type of operation is flexible, and the three-dimensional adjustment of the metatarsal bone can be performed up and down, forward and backward, and inward and outward. Besides, the “gap area” left by the wedge-shaped osteotomy leaves more buffer for the rotation of the metatarsals during the operation, which could ensure the curative effect.

The long osteotomy line of the metatarsal diaphysis in MRSO can be extended to nearly the full length of the metatarsal bone (Fig. 3). Its longer osteotomy line than TSO gives it greater ability to correct deformities. The distal osteotomy line of the MRSO is carried out in the cancellous bone of the metaphysis. As stated in other studies, troughing can be prevented by placing resected cortical bone between the gap sites created by osteotomy [19, 40]. The distal osteotomy line of the MRSO is carried out in the metaphyseal cancellous bone and the “gap” (the dorsal medial side of the distal metatarsal after rotation) was filled by the spare wedge-shaped bone piece, so that the cortical bone at the osteotomy end could be pressed each other to form a “lock” after rotation, which can effectively prevent the troughing effect once the osteotomy is displaced [41]. Meanwhile, the “lock” structure, could ensures the stability after osteotomy. In MRSO group, the distal end of the osteotomy line was about 20 mm from the metatarsophalangeal joint surface; the proximal end was about 5 mm from the tarsometatarsal joint, which ensured the length and stability of the osteotomy shaft. There was no “troughing effect” in this group throughout the follow-up.

In order to achieve better radiological performance, all the MRSO operations were combined with Akin osteotomy for the medium to serious hallux valgus in the MRSO group. The application of Akin osteotomy is increasingly recognized [42–45]. Akin osteotomy can effectively improve the increase of HVA and effectively compensate for the metatarsal osteotomy, thus significantly improving the clinical efficacy and patient satisfaction rate, and fully making up for the shortcomings of rotary Scarf osteotomy in phalanx deformities [10].

MRSO could aid in the recovery of gait biomechanical function post-surgery for the more stable broken ends [37] in MRSO allows for more adequate recovery of foot muscle strength during postoperative functional exercises, thereby restoring normal gait. Although there was no statistical difference in the final AOFAS scores between the two groups, the MRSO group was slightly better at final follow-up.

The strength of this study is that the institution to which this study belongs is the foot and ankle center of this city, thus sufficient eligible cases have been accumulated within 4 years. All surgeries in this study were performed by one single senior experienced surgeon who specialized in foot and ankle surgery. However, backward-looking clinic trial was a limitation in our study, and the planning and arrangement are not as detailed as forward-looking clinic trial. In this clinical trial, the standard deviation in the sample data regarding HV especially after surgery is large, and it is inevitable that the intra-observer reliability will decrease. Since data collection was completed by a single researcher, different researchers will be arranged to conduct measurements in future clinical work, and each of them will repeat the measurements multiple times to improve inter-observer and intra-observer reliability. Besides, more and more scholars pay attention to the impact of hypermobility of the first ray (HFR) and instability of the First Metatarsal-Cuneiform Joint (I-MTCJ) on HV [46]. HV patients with similar HVA and IMA angles but different degrees of HFR and I-MTCJ may have different prognosis after the same rotational Scarf surgery. In view of this, our study lacked the evaluation of HFR and I-MTCJ, and we will take these two factors into consideration as observation indicators in future studies. After all, twenty-four months is a short period, and a longer follow-up period would certainly improve the usefulness of the study. In addition, the preoperative, postoperative, and follow-up clinical and radiographic data were evaluated by only one investigator.

Above all, MRSO showed comparable results to TSO in terms of HVA, AOFAS and MTP-1 ROM, but MRSO showed significant advantages in improving IMA, sesamoid grade. In spite of aggravating the DMAA, MRSO can correct hallux valgus deformity in three dimensions, meets the ideal requirements of foot orthosis, and has a low complication rate.

## Conclusion

In summary, MRSO showed comparable results to TSO, and improved IMA and sesamoid grade to a greater extent, with a lower probability of through effect. Despite the increase of DMAA, MRSO still is a reproducible, non-dangerous and efficacious alternative technique for HV treatment.

## Data Availability

For data requests, please contact the author Fang Zhenhua.

## Abbreviations

TSO: Traditional Scarf osteotomy
MRSO: Modified Rotary Scarf osteotomy
MSHV: Moderate to Severe Hallux Valgus
HVA: Hallux Valgus Angle
MTP-1 ROM: first Metatarsophalangeal joint Range of Motion
IMA: Intermetatarsal Angle
DMAA: Distal metatarsal articular angle
AOFAS: American Orthopaedic Foot and Ankle Society
CORA: center of rotation of angulation
